# Supplementary material for: Curvicollide D Isolated from the Fungus Amesia sp. Kills African Trypanosomes by Inhibiting Transcription
Source: Int J Mol Sci. 2022 May 29;23(11):6107. doi: 10.3390/ijms23116107 (PMC9181715; doi:10.3390/ijms23116107)
Supplement: Supplementary file 1 [file ijms-23-06107-s001.zip › ijms-1728148-supplementary/SupInfo Ortiz-Gonzalez et al..pdf]

Supplementary Information

Curvicollide D Isolated from the Fungus *Amesia* sp. Kills African Trypanosomes by Inhibiting Transcription

Matilde Ortiz-Gonzalez [1,2], Ignacio Pérez-Victoria [3], Inmaculada Ramirez-Macias [2,4], Nuria de Pedro [3], Angel Linde-Rodriguez [2,4], Víctor González-Menéndez [3], Victoria Sanchez-Martin [2,4], Jesús Martín [3], Ana Soriano-Lerma [2,5], Olga Genilloud [3], Virginia Perez-Carrasco [2,4], Francisca Vicente [3], JoséMaceira [2], Carlos A Rodrígues-Poveda [2], JoséMaría Navarro-Marí [4], Fernando Reyes [3,*], Miguel Soriano [1,2,†] and Jose A. Garcia-Salcedo [2,4,*,†]

1 Center for Intensive Mediterranean Agrosystems and Agri-Food Biotechnology (CIAIMBITAL), University of Almeria, 04001 Almería, Spain; mog042@ual.es (M.O.-G.); msoriano@ual.es (M.S.)
2 GENYO, Centre for Genomics and Oncological Research: Pfizer, University of Granada, Andalusian Regional Government, PTS Granada-Avenida de la Ilustración, 18016 Granada, Spain; inmaculada.ramirez@genyo.es (I.R.-M.); angel.linde@genyo.es (A.L.-R.); victoria.sanchez@genyo.es (V.S.-M.); ana.soriano@genyo.es (A.S.-L.); virginia.perez@genyo.es (V.P.-C.); pemaceira@gmail.com (J.M.); crodriguespoveda@gmail.com (C.A.R.P.)
3 Fundación MEDINA, Centro de Excelencia en Investigación de Medicamentos Innovadores en Andalucía, Avenida del Conocimiento 34, 18016 Granada, Spain; ignacio.perez-victoria@medinaandalucia.es (I.P.-V.); ndepedro@lifelength.com (N.d.P.); victor.gonzalez@medinaandalucia.es (V.G.-M.); jesus.martin@medinaandalucia.es (J.M.); olga.genilloud@medinaandalucia.es (O.G.); francisca.vicente@medinaandalucia.es (F.V.)
4 Microbiology Unit, Biosanitary Research Institute IBS.Granada, University Hospital Virgen de las Nieves, 18014 Granada, Spain; josem.navarro.sspa@juntadeandalucia.es
5 Department of Physiology, Faculty of Pharmacy, Campus Universitario de Cartuja, Institute of Nutrition and Food Technology "José Mataix", University of Granada, 18071 Granada, Spain
* Correspondence: fernando.reyes@medinaandalucia.es (F.R.); jags@genyo.es (J.A.G.-S.); Tel.: +34-958993965 (F.R.); +34-958715500 (J.A.G.-S.)
† These authors contributed equally to this work.

List of Supplementary Materials:

Figure S1. UV (DAD) spectrum of curvicollide D.

Figure S2. ESI-TOF HRMS spectrum of curvicollide D.

Figure S3. ^{1}H NMR spectrum, showing expanded regions, of curvicollide D (500 MHz, $CDCl_3$, 24 °C).

Figure S4. ^{13}C NMR spectrum, showing expanded regions, of curvicollide D (125 MHz, $CDCl_3$, 24 °C).

Figure S5. COSY spectrum of curvicollide D.

Figure S6. TOCSY spectrum of curvicollide D.

Figure S7. NOESY spectrum of curvicollide D.

Figure S8. Multiplicity edited HSQC spectrum of curvicollide D.

Figure S9. HMBC spectrum of curvicollide D.

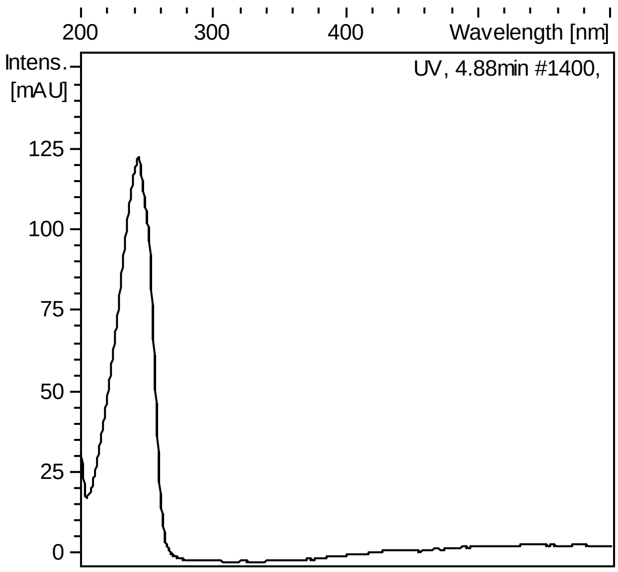

Figure S1. UV (DAD) spectrum of curvicollide D.

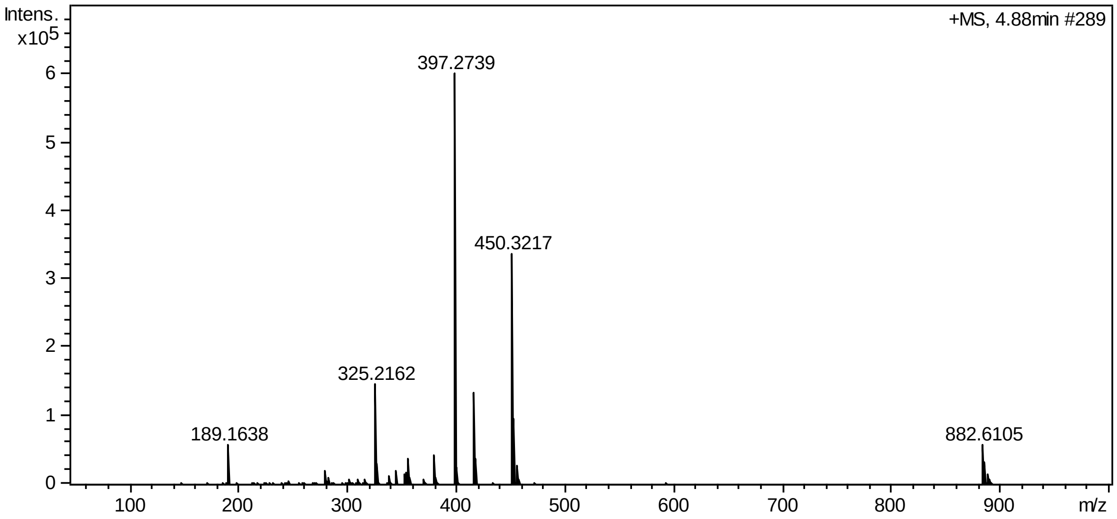

Figure S2. (+)-ESI-TOF HRMS spectrum of curvicollide D.

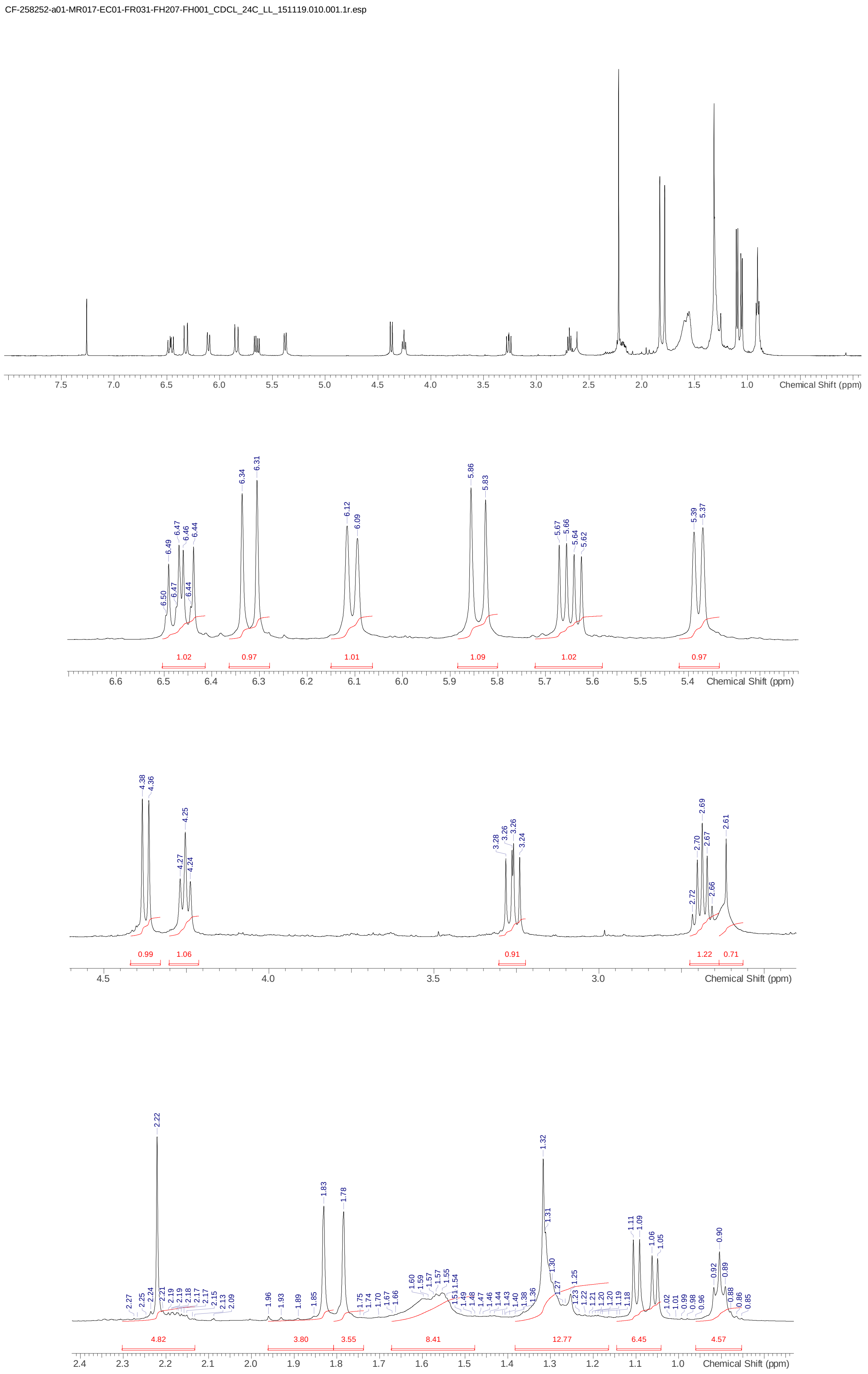

Figure S3. ^{1}H NMR spectrum, showing expanded regions, of curvicollide D (500 MHz, $CDCl_3$, 24 °C).

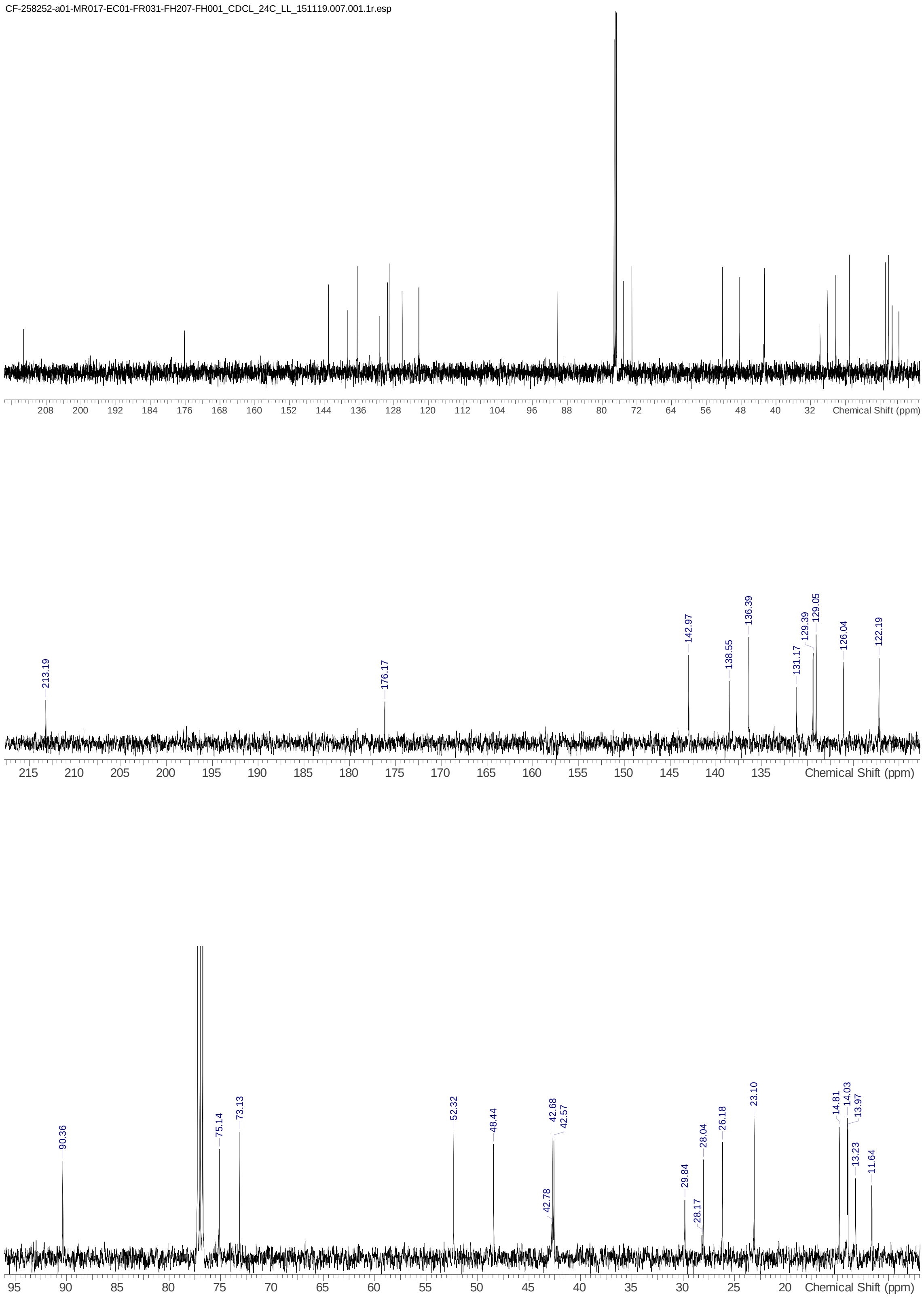

Figure S4. ^{13}C NMR spectrum, showing expanded regions, of curvicollide D (125 MHz, $CDCl_3$, 24 °C).

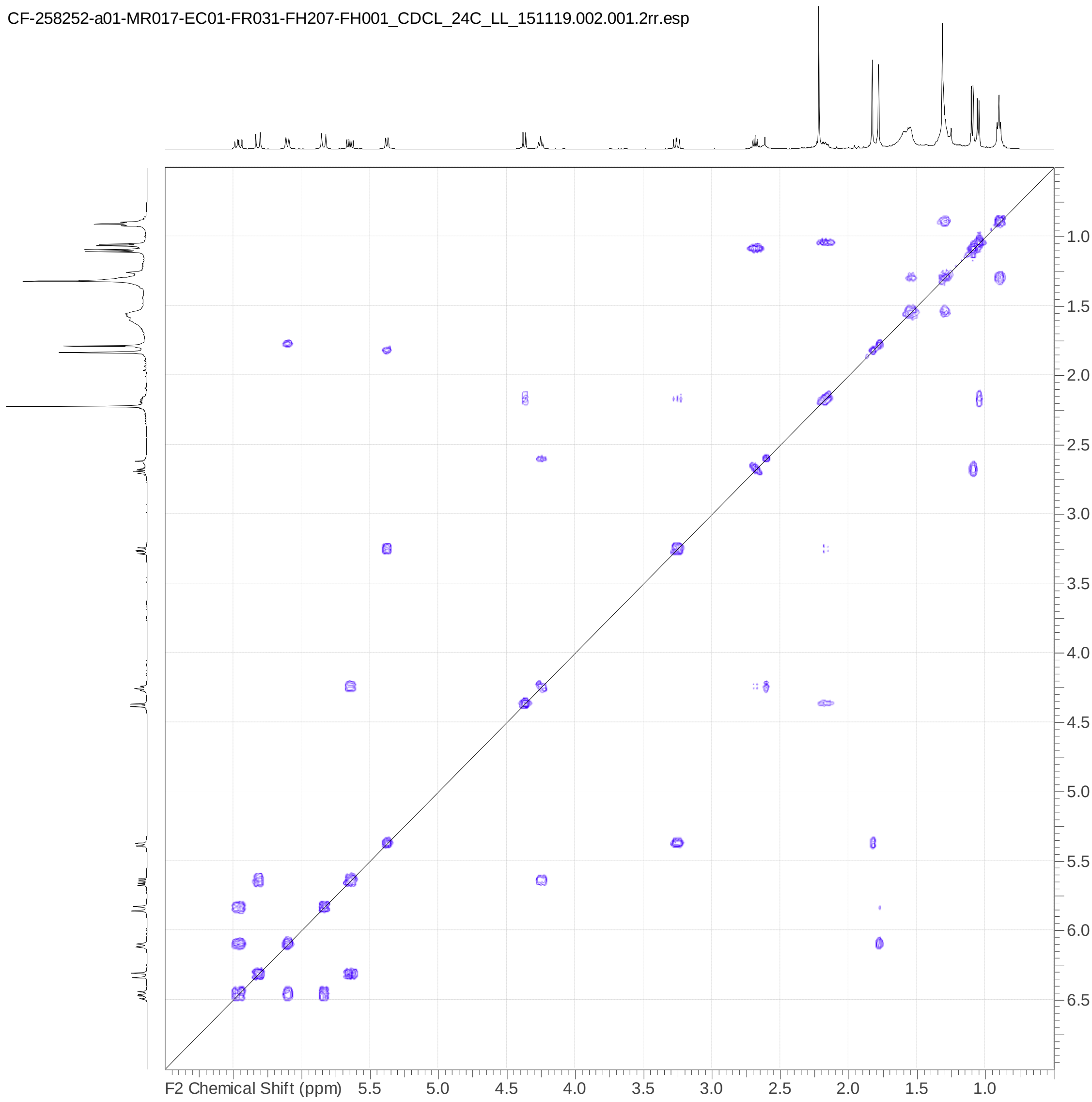

Figure S5. COSY spectrum of curvicollide D.

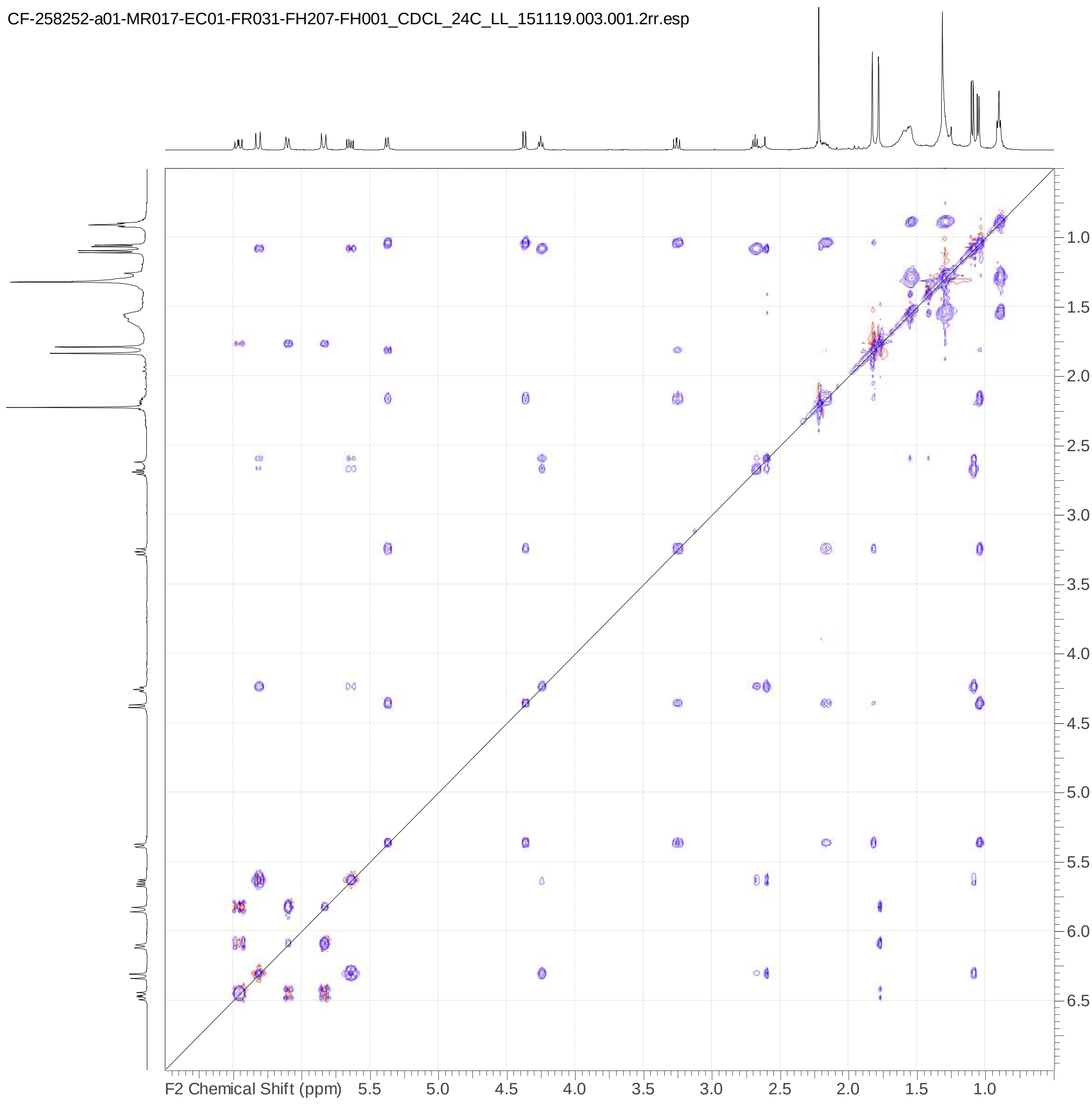

Figure S6. TOCSY spectrum of curvicollide D.

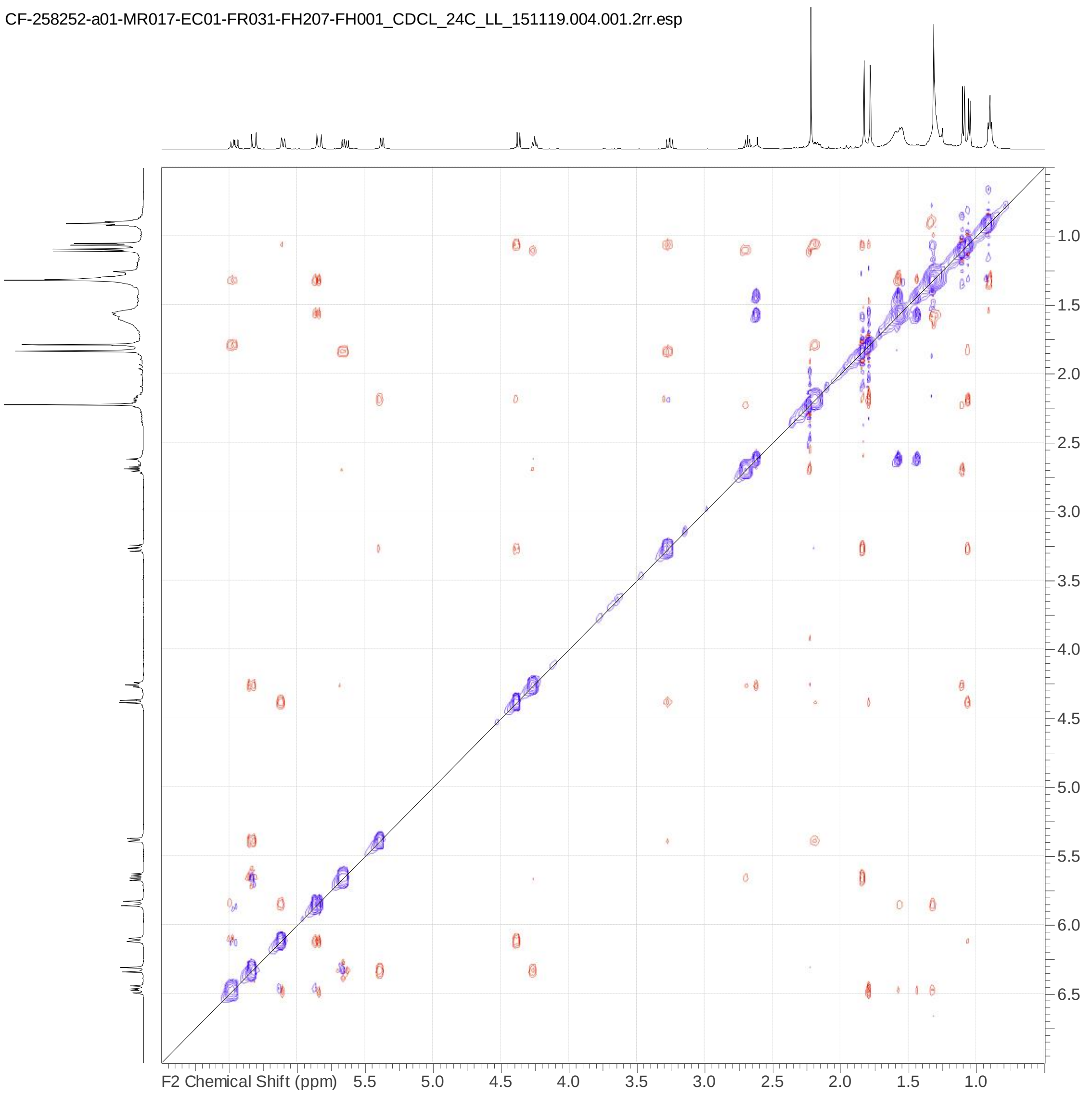

Figure S7. NOESY spectrum of curvicollide D.

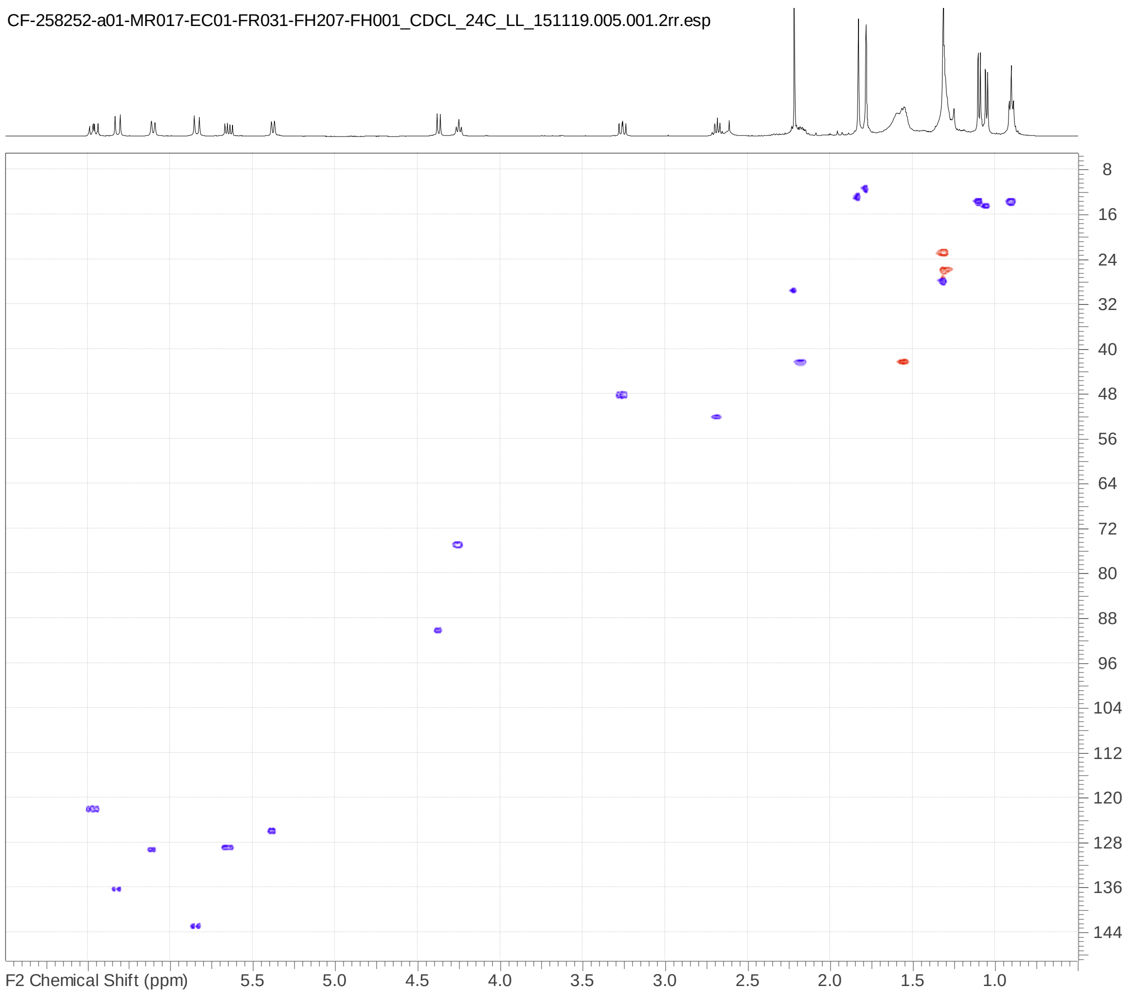

Figure S8. Multiplicity edited HSQC spectrum of curvicollide D.

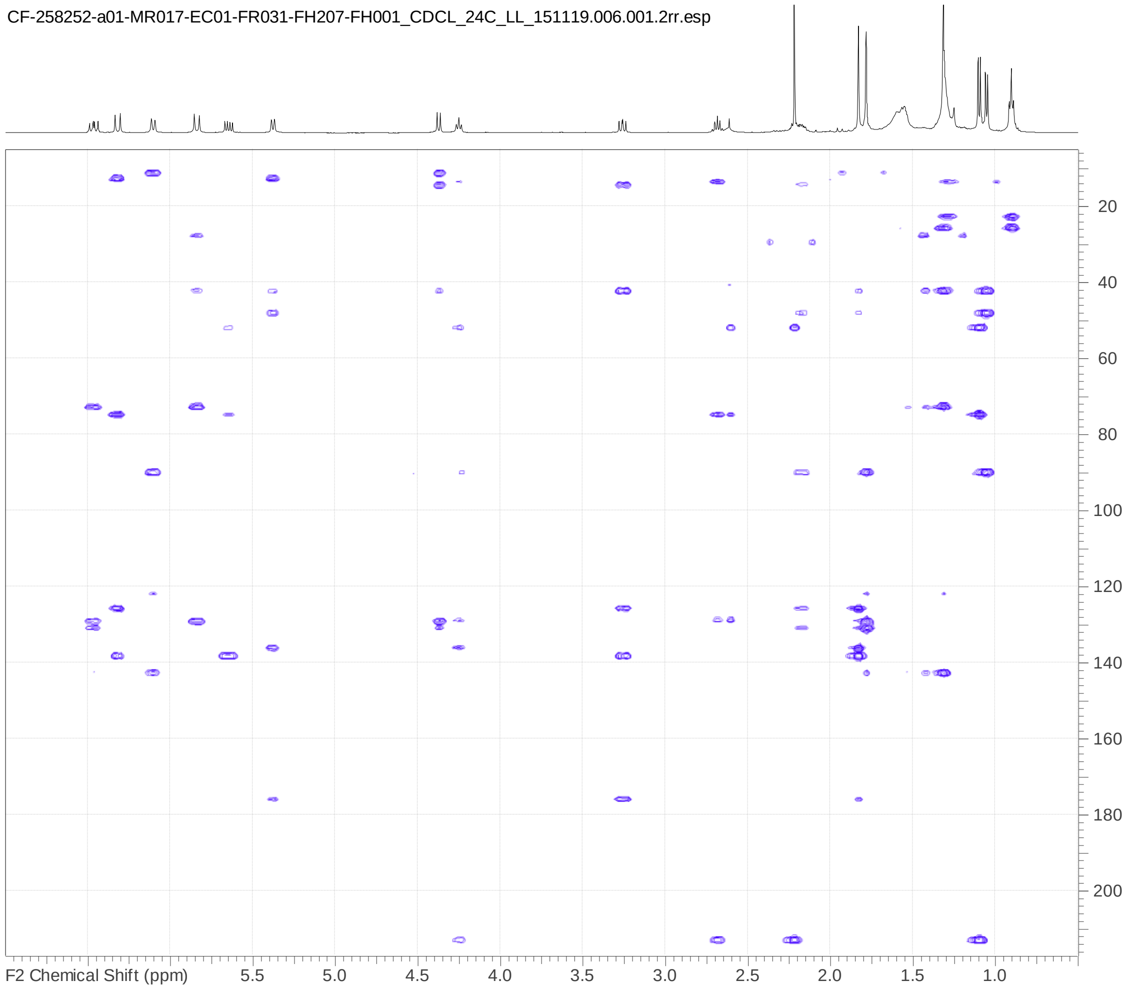

Figure S9. HMBC spectrum of curvicollide D.
